# Reply to Jue, J.S.; Alameddine, M. Role of PSA Density and MRI in PSA Interpretation. Comment on “Lumbreras et al. Variables Associated with False-Positive PSA Results: A Cohort Study with Real-World Data. *Cancers* 2023, *15*, 261”

**DOI:** 10.3390/cancers15102685

**Published:** 2023-05-10

**Authors:** Blanca Lumbreras, Lucy Anne Parker, Juan Pablo Caballero-Romeu, Luis Gómez-Pérez, Marta Puig-García, Maite López-Garrigós, Nuria García, Ildefonso Hernández-Aguado

**Affiliations:** 1 Department of Public Health, University Miguel Hernández de Elche, 03550 Alicante, Spain; lparker@umh.es (L.A.P.); marta.puig@alu.umh.es (M.P.-G.); ihernandez@umh.es (I.H.-A.); 2 CIBER de Epidemiología y Salud Pública (CIBERESP), 28029 Madrid, Spain; lopez_marter@gva.es; 3 Department of Urology, University General Hospital of Alicante, 03010 Alicante, Spain; juanpablocaballero@gmail.com (J.P.C.-R.); ngkarb@gmail.com (N.G.); 4 Alicante Institute for Health and Biomedical Research (ISABIAL), 03010 Alicante, Spain; 5 Urology Department, General University Hospital of Elche, 03203 Elche, Spain; l.gomez@umh.es; 6 Pathology and Surgery Department, Miguel Hernández University of Elche, 03550 Alicante, Spain; 7 Clinical Laboratory Department, University Hospital of San Juan de Alicante, Sant Joan d’Alacant, 03550 Alicante, Spain

We thank you and your co-authors for the comment [1] on our article “Variables Associated with False-Positive PSA Results: A Cohort Study with Real-World Data” published in *Cancers* [2]. We have read your comments with great interest on the use of other parameters that could increase the accuracy of a prostate cancer diagnosis.

Screening for prostate cancer with PSA remains a controversial topic due to its relatively low specificity and the subsequent risk of overdiagnosis and overtreatment. Several studies have evaluated the implementation of new parameters to improve the detection of prostate cancer. However, most of these studies [3] identified biomarkers to reduce unnecessary biopsies in patients with suspected prostate cancer due to high PSA levels, and therefore, they cannot be considered screening tests. For instance, PSA density could be considered a better biomarker for predicting prostate cancer compared with PSA alone, but the main conclusion of a previous study was that the use of PSA density could help avoid biopsies in men whose PSA was elevated [4]. Moreover, PSA density depends on the measurement of the prostate volume, which can be highly variable [5]. In our study, we aimed to evaluate real world-data in primary care on the predictive value of PSA testing as an opportunistic screening tool or as a diagnostic test in patients with suspected symptoms of prostate cancer. That is why we excluded patients with previously elevated PSA results from our study.

In 2021, The European Association of Urology developed a risk-adapted strategy [6] that could be useful in reducing the probability of overdiagnosis and overtreatment since it could help to differentiate between the likelihood of no prostate cancer, indolent prostate cancer, and aggressive prostate cancer. In accordance with these recommendations, PSA testing should be offered to well-informed men with an elevated risk of prostate cancer as part of this risk-adapted strategy which also includes risk calculators, multi-parametric magnetic resonance imaging (mpMRI) and biomarker tests. Nevertheless, opportunistic screening for prostate cancer is usually carried out in primary care, where general practitioners order PSA on the basis of a patient’s risk factors and preferences, and as such, they do not usually have other test results to consider at the time of petition. The use of diagnostic tests such as mpMRI or PSA density is limited to hospital settings for patients with elevated PSA results.

Recent studies have demonstrated how mpMRI can predict the presence of clinically significant prostate cancer with a sensitivity of 93%, and thus, its use as a triage test before the first prostate biopsy could reduce unnecessary biopsies by 25% [7]. In our study, those men with an elevated PSA level had a transrectal ultrasound-guided biopsy to confirm the diagnosis suspicion. Our study was carried out in 2018 when the recommendations regarding the mpMRI were not established in practice. In addition, the interpretation of mpMRI was highly variable depending on the experience of the radiologist and the center where it was applied [8]. Therefore, although it increased specificity in the diagnosis of prostate cancer, its application in routine practice was less clear. However, the use of mpMRI or the transrectal ultrasound-guided biopsy did not influence the results of our study since we evaluated the false-positive PSA results of opportunistic screening, not the results of the final diagnosis of prostate cancer.

We completely agree with the authors that efforts to increase the specificity in the detection of prostate cancer should be made. In addition, according to available evidence, the determination of PSA density and mpMRI in patients with elevated PSA could decrease the probability of overdiagnosis and overtreatment. Nevertheless, there is a large number of PSA determinations carried out in primary care, which could be associated with adverse results. We think that the evaluation of those factors associated with the probability of a false-positive PSA result could support general practitioners’ clinical decision making.
